# Allotetraploid Origin and Putative Ancient Introgression in *Plantago hakusanensis* (Plantaginaceae)

**DOI:** 10.1002/ece3.71144

**Published:** 2025-03-17

**Authors:** Naoko Ishikawa, Shota Sakaguchi, Chikako Hasekura, Alexey Shipunov, Ayumi Matsuo, Yoshihisa Suyama, Hirokazu Tsukaya, Hiroshi Ikeda, Motomi Ito

**Affiliations:** ^1^ Kawatabi Field Science Center, Graduate School of Agricultural Science Tohoku University Osaki Miyagi Japan; ^2^ Graduate School of Human and Environmental Studies Kyoto University, Yoshida‐Nihonmatsu‐Cho Kyoto Japan; ^3^ Faculty of Agriculture Tokyo University of Agriculture Atsugi Kanagawa Japan; ^4^ Department of Biology Minot State University Minot North Dakota USA; ^5^ GENODAS Inc Urbannet Sendai‐Chuo Bld Sendai Miyagi Japan; ^6^ Graduate School of Science The University of Tokyo Tokyo Japan; ^7^ The University Museum The University of Tokyo Tokyo Japan; ^8^ Graduate School of Arts and Sciences the University of Tokyo Tokyo Japan

**Keywords:** allopolyploid, chloroplast capture, endangered species, introgression, phylogeographical inference, *Plantago*

## Abstract

*Plantago hakusanensis* (2*n* = 4*x* = 24) is an endangered endemic species that occurs in subalpine zones in Japan. To clarify the unresolved phylogenetic position of *P. hakusanensis* within subgenus *Plantago*, we constructed a phylogenetic tree based on the nuclear‐encoded single‐copy gene sucrose–proton symporter 1 (*SUC1*) using 60 previously reported alleles from 24 taxa in subgenus *Plantago*. We found that *P. hakusanensis* was closely related to *Plantago asiatica* var. *densiuscula*. The phylogenetic relationships between *P. hakusanensis* and *P. asiatica* var. *densiuscula* were further examined by analyses of the *SUC1* nuclear regions and the internal transcribed spacer (ITS) of rDNA, genome‐wide single‐nucleotide polymorphism genotyping (via multiplexed inter‐simple sequence repeat genotyping by sequencing), as well as by additional analyses of three chloroplast (cp) regions (*trnL‐F*, *ndhF‐rpl32*, and *rpl32‐trnL*) in 25 individuals of *P. hakusanensis* and 53 individuals of *P. asiatica* var. *densiuscula* covering the species' geographical distribution. Monophyly of *P. hakusanensis* was suggested by the nuclear marker analyses, whereas the cp haplotypes of *P. hakusanensis* were shared with *P. asiatica* var. *densiuscula* and *P. asiatica* in China. The disparity between the nuclear and cp data may be explained by the introgression of the cp genome (cp capture). This research provides a phylogenetic tree showing the position of *P. hakusanensis* within subgenus *Plantago* and molecular evidence that implies complicated introgressions between *P. hakusanensis* and *P. asiatica* var. *densiuscula*.

## Graphical abstract

*Plantago hakusanensis* (2*n* = 4*x* = 24) is an endangered endemic species in subalpine zones in Japan. *P. hakusanensis* is closely related to *P. asiatica* var. *densiuscula*, and each species is monophyletic in terms of nuclear markers, but the chloroplast haplotypes are shared. This disparity may be indicative of chloroplast genome introgression (chloroplast capture) between the two species.

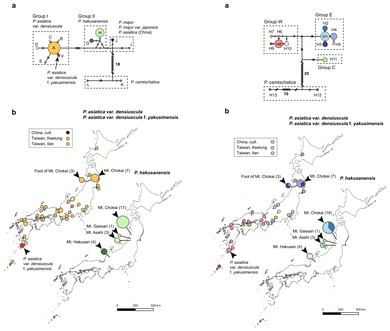

## 1 Introduction

*Plantago hakusanensis* Koidz. (Plantaginaceae) (2*n* = 4*x* = 24) is a perennial herb endemic to Japan (Yamazaki 1993). *P. hakusanensis* is distributed around snow patches and wet fields in subalpine zones in Honshu (*ca*. 1500–2300 m above sea level; Koizumi 1930; Yamazaki 1992, 1993). The species has been found on 12 mountains located between Mt. Moriyoshi (Akita Prefecture) to the north and Mt. Hakusan (Ishikawa Prefecture) to the south (Yamada and Satomi 1975). *P. hakusanensis* and its hairless form, *P. hakusanensis* f. *glabra* T. Yamaz., have been recognized as category I endangered taxa in Akita and Nagano Prefectures, and as category II vulnerable taxa in Fukushima, Gunma, Ishikawa, and Gifu Prefectures (Japanese Red Data 2020). Based on morphological features, *P. hakusanensis* has been assigned to the subgenus *Plantago*, which comprises *ca*. 131 species in five sections (Rahn 1996). Rahn (1996) suggested that *P. hakusanensis* belongs to sect. *Plantago* and is related to *P. asiatica* L. However, molecular phylogenetic studies within this subgenus (Ishikawa et al. 2009) have shown that the sect. *Plantago* is paraphyletic and requires reclassification, and no molecular phylogenetic analyses have been conducted to identify the species most closely related to *P. hakusanensis*. In addition, the sectional placement of *P. hakusanensis* has been debated by some researchers. For example, Yamazaki (1992) suggested that *P. hakusanensis* is most closely related to *P. gentianoides* and placed it in section *Gentianoides* Pilg. because it possesses anthers with a slender acuminate projection at the apex and seeds with a concave ventral face, as observed in *Plantago gentianoides* Sibth. et Smith from central Asia.

*P. hakusanensis* has been identified as a conservation target on Mt. Hakusan because of concerns that it may be genetically polluted via hybridization with *P. asiatica* L. var. *densiuscula* Pilg. (2*n* = 4*x* = 24) (Pilger 1922; Nogami 2001; Nakayama et al. 2006, 2008; Sano et al. 2016, 2019). *P. asiatica* var. *densiuscula* is a perennial herb distributed from the Japanese Archipelago to Taiwan (Pilger 1922). It commonly grows in sunny locations, such as roadsides and unpaved parking lots, at low elevations (Pilger 1922; Yamazaki 1993; Ishikawa et al. 2006, 2009). *P. asiatica* var. *densiuscula* is morphologically similar to *P. hakusanensis* , but the two taxa differ in several respects, including the numbers of seeds per fruit, seed morphology, and leaf shape. *P. hakusanensis* has one to two seeds per fruit, whereas *P. asiatica* var. *densiuscula* has four to seven seeds per fruit (Pilger 1922; Yamazaki 1993; Nakayama et al. 2008; Ohashi 2017). *P. asiatica* var. *densiuscula* has invaded the native habitats of *P. hakusanensis* on Mt. Hakusan, such as Minami Ryugabanba Campsite (approximately 2080 m above sea level) (Nogami 2001, 2002, 2003; Nakayama et al. 2005, 2008; Sano et al. 2022). The sticky wet seeds of *P. asiatica* var. *densiuscula* may have been carried to the subalpine zone via the shoes of hikers or along with construction materials of mountain shelters (Nogami 2001, 2003). The two taxa are genetically compatible, and fertile F1 hybrids have been obtained by artificial pollination (Sano et al. 2016). The occurrence of protogynous and wind‐pollinated flowers in the two taxa increases their outcrossing rate; moreover, they had overlapping flowering periods on Mt. Hakusan during 3 out of the 4 years between 2011 and 2014 (Sano et al. 2019). Putative hybrids with an intermediate leaf shape have been found on Mt. Hakusan at locations where the taxa are sympatric (Nakayama et al. 2008). The government ministries of Japan (Ministry of Agriculture, Forestry and Fisheries of Japan et al. 2015) led efforts to remove *P. asiatica* var. *densiuscula* from Mt. Hakusan, where it is regarded as an exotic taxon. Genetic pollution is also a concern in other locations. Invasions of *P. asiatica* var. *densiuscula* have been reported on Mt. Gassan (Yokoyama 2015). Although *P. hakusanensis* is (i) an endemic species with limited distribution and (ii) threatened by hybridization with *P. asiatica* var. *densiuscula*, its phylogenetic relationships and evolutionary origins have not been investigated adequately.

Polyploidy is frequent in the subgenus *Plantago* (67% of species, as shown by chromosome counts, Rahn 1996), and allopolyploidy has been identified by molecular phylogenetic evidence (Ishikawa et al. 2009). Hence, we aimed to determine the phylogenetic position of tetraploid *P. hakusanensis* within the subgenus *Plantago* via phylogenetic analyses based on the nuclear‐encoded single‐copy gene sucrose–proton symporter 1 (*SUC1*) using 60 previously reported alleles from 24 representative taxa in the subgenus *Plantago*. We obtained the DNA sequence of *SUC1* in *P. hakusanensis* using either cloning or allele (homoeolog)‐specific PCR amplification. Furthermore, although Mt. Chokai is one of the popular destinations among hikers during the summer, there have been no previous reports of an invasion by *P. asiatica* var. *densiuscula*. To investigate the potential invasion of *P. asiatica* var. *densiuscula* into subalpine areas and to assess possible hybridization between *Plantago hakusanensis* and *P. asiatica* var. *densiuscula* on Mt. Chokai, we conducted phylogenetic analyses of the nuclear‐encoded rDNA internal transcribed spacer (ITS) regions and performed genome‐wide single‐nucleotide polymorphism (SNP) genotyping using multiplexed inter‐simple sequence repeat genotyping by sequencing (MIG‐seq) (Suyama and Matsuki 2015; Suyama et al. 2022) and three chloroplast (cp) regions (*trn*L‐F, *ndh*F‐*rpl*32, and *rpl*32‐*trn*L).

## 2 Materials and Methods

### 2.1 Taxon Sampling and DNA Isolation

To determine the phylogenetic position of *Plantago hakusanensis* within the subgenus *Plantago*, we conducted a phylogenetic analysis using a nuclear‐encoded, single‐copy *SUC1* gene region spanning from exon 1 to exon 2. We newly sequenced this region from two individuals of *P. hakusanensis* collected from Mt. Hakusan and incorporated 60 previously reported *SUC1* alleles from 24 taxa (Ishikawa et al. 2009; Table 1). These taxa represent all five sections (*Micropsyllium*, *Mesembrynia*, *Virginica*, *Oliganthos*, and *Plantago*) of the globally distributed subgenus *Plantago* (Rahn 1996). Alleles were isolated from one individual of each of these 24 taxa, except in the case of diploid *P. major* L., from which we collected two individuals. The numbers of alleles obtained from each individual varied from one to seven, depending on the levels of ploidy and/or heterozygosity (Ishikawa et al. 2009). We selected *P.* *tenuiflola* Waldst. & Kit, and one of the 24 taxa evaluated by Ishikawa et al. (2009) as an outgroup based on previously determined phylogenetic relationships within subgroup *Plantago* (Rønsted et al. 2002; Ishikawa et al. 2009; Iwanycki Ahlstrand et al. 2019). Note that *Plantago formosana* Tateishi & Masam. is considered a synonym of *P. asiatica* subs *P.* *asiatica* and has been discussed as a synonym of *Plantago major* L. (Hatusima 1971; Shimabuku 1997). The phylogenetic analysis showed that *P. hakusanensis* is closely related to *P. asiatica* var. *densiuscula*. Therefore, to investigate differences between *P. hakusanensis* and *P. asiatica* var. *densiuscula* further, we conducted additional analyses using genetic polymorphisms obtained from the same *SUC1* region (extending from exon 1 to exon 2), the nuclear‐encoded rDNA ITS regions, and three cp regions (*trnL‐F*, *ndhF‐rpl32*, and *rpl32‐trnL*). We also used MIG‐seq to identify genome‐wide SNPs (Suyama and Matsuki 2015; Suyama et al. 2022). We used 25 individuals of *P. hakusanensis* and 53 individuals of *P. asiatica* var. *densiuscula*, including *P. asiatica* var. *densiuscula* f. *yakusimensis* (Masam) N. Ishikawa et al. The samples of *P. hakusanensis* were collected from Mt. Hakusan (4 individuals), Mt. Chokai (3 populations, 17 individuals), Mt. Gassan (1 individual), and Mt. Asahi (3 individuals), thereby covering the main distribution range of the taxon. Mt. Hakusan is the lectotype locality, and both Mt. Chokai and Mt. Gassan were listed as known localities of *P. hakusanensis* specimens in the original description (Koizumi 1930). The samples of *P. asiatica* var. *densiuscula* comprised 46 individuals collected from Taiwan, Cheju Island in Korea, and a broad geographical range across the Japanese Archipelago. We also included seven individuals from the subalpine zone on Mt. Chokai to investigate hybridization between *P. hakusanensis* and *P. asiatica var*. *densiuscula* invaders in the subalpine zone. Although *P. hakusanensis* was primarily distributed around snow patches and *P. asiatica* var. *densiuscula* was observed near both former and current mountain huts (Table 2), the distributions of the two taxa overlapped along a mountain trail on Mt. Chokai at elevations of 1320–1540 m.

**TABLE 1:** List of 24 taxa used for the *SUC1* phylogenetic analysis to determine the phylogenetic position of *P. hakusanensis* .

No.	Section	Species	Number of allele (s)	References (sample name)
1	*Plantago*	*P. cornuti* Gouan.	1	Ishikawa et al. (2009)
2		*P. major* L.	1	Ishikawa et al. (2009), (Japan)
		*P. major*	1	Ishikawa et al. (2009), (Germany)
3		*P. major* var. *japonica* (Franch. et Sav.) Miyabe	1	Ishikawa et al. (2009)
4		*P. maxima* Jacq.	2	Ishikawa et al. (2009)
5		*P. reniformis* Beck.	1	Ishikawa et al. 2009
6		*P. asiatica* L. var. *densiuscula* Pilg.	2	Ishikawa et al. (2009)
7		*P. asiatica* var. *densiuscula* f. *yakusimensis* (Masam.) N. Ishikawa et al.	2	Ishikawa et al. (2009)
8		*P. palmata* Hook. f.	2	Ishikawa et al. (2009)
9		*P. rugelii* Decne.	2	Ishikawa et al. (2009)
10		*P. formosana* Tateishi et Masam.	3	Ishikawa et al. (2009)
11		*P. media* L.	4	Ishikawa et al. (2009)
12	*Mesembrynia*	*P. debilis* R. Br.	1	Ishikawa et al. (2009)
13		*P. depressa* Willd.	1	Ishikawa et al. (2009)
14		*P. camtschatica* Link.	1	Ishikawa et al. (2009)
15		*P. stauntoni* Reichardt.	2	Ishikawa et al. (2009)
16		*P. spathulata* Hook. f.	7	Ishikawa et al. (2009)
17		*P. raoulii* Decne.	4	Ishikawa et al. (2009)
18	*Virginica*	*P. tomentosa* Lam.	2	Ishikawa et al. (2009)
19		*P. trinitatis* Rahn.	2	Ishikawa et al. (2009)
20		*P. virginica* L.	2	Ishikawa et al. (2009)
21		*P. australis* Lam.	4	Ishikawa et al. (2009)
22	*Oliganthos*	*P. uniglumis* Walp.	5	Ishikawa et al. (2009)
23		*P. rigida* Kunth.	5	Ishikawa et al. (2009)
24	*Micropsyllium*	*P. tenuiflola* Waldst. et Kit	2	Ishikawa et al. (2009)

**TABLE 2:** The information of the taxa and populations analyzed in this study.

Population no.	Sample size	ITS genotype (*n*)	Cp haplotype (*n*)	Latitude, longitude	Altitude (m)	Locality	Reference
*P. hakusanensis*							
1	3	H (3)	H1 (1), H11 (2)	Unknown	Unknown	Mt. Asahi, Asahi‐cho, Nishimurayama‐gun, Yamagata pref., Japan	
2	9	H (9)	H1 (4), H2 (5)	39°04′ N, 140°02′ E	1556	Mt. Chokai, Yusa‐cho, Akumi‐gun, Yamagata pref., Japan	
3	5	H (5)	H1 (3), H2 (1)	39°04′ N, 140°02′ E	1517	Mt. Chokai, Yusa‐cho, Akumi‐gun, Yamagata pref., Japan	
4	3	H (3)	H1 (3)	39°04′ N, 140°02′ E	1282	Mt. Chokai, Yusa‐cho, Akumi‐gun, Yamagata pref., Japan	
5	1	H (1)	H11 (1)	38°32′ N, 139°59′ E	1388	Mt. Gassan, Tsuruoka, Yamagata pref., Japan	
6	4	G (4)	H10 (4)	36°08′ N, 136°46′ E	2072	Mt. Hakusan, Shiramine, Hakusan city, Ishikawa pref., Japan	
*P. asiatica* var. *densiuscula*							
7	4	A (4)	H3 (4)	39°04′ N, 140°02′ E	1539	Mt. Chokai, Yusa‐cho, Akumi‐gun, Yamagata pref., Japan	
8	3	A (3)	H4 (2), H8 (1)	39°04′ N, 140°02′ E	1282	Mt. Chokai, Yusa‐cho, Akumi‐gun, Yamagata pref., Japan	
9	3	A (3)	H3 (2), H4 (1)	39°06′ N, 139°52′ E	4	Fukuura Misaki, Yusa‐cho, Akumi‐gun, Yamagata pref., Japan	
10	2	A (1)	H8 (1)	34°40′ N, 135°50′ E	112	Nara park, Nara pref., Japan	Ishikawa et al. (2006)^a^
11	1	ND	H8 (1)	34°39′ N, 135°50′ E	107	Nara University of Education, Nara pref., Japan	
12	1	A (1)	H1 (1)	Unknown	Unknown	Shirakawa, Gifu pref., Japan	Ishikawa et al. (2006)^a^
13	2	A (1)	H8 (1)	35°00′ N, 135°51′ E	123	Otu city, Shiga pref., Japan	Ishikawa et al. (2006)^a^
14	1	A (1)	ND	35°00′ N, 135°47′ E	64	Sakyo‐ku, Kyoto pref., Japan	Ishikawa et al. (2006)^a^
15	1	A (1)	H4 (1)	34°27′ N, 136°43′ E	18	Ise city, Mie pref., Japan	Ishikawa et al. (2006)^a^
16	1	A (1)	H8 (1)	34°14′ N, 133°00′ E	6	Ohmi‐shima Island, Ehime pref., Japan	Ishikawa et al. (2006)^a^
17	1	A (1)	H6 (1)	33°53′ N, 133°11′ E	44	Saijyo city, Ehime pref., Japan	Ishikawa et al. (2006)^a^
18	1	A (1)	ND	33°35′ N, 130°24′ E	3	Fukuoka city, Fukuoka pref., Japan	Ishikawa et al. (2006)^a^
19	2	A (2)	H8 (1)	33°31′ N, 130°32′ E	50	Dazaifu city, Fukuoka pref., Japan	Ishikawa et al. (2006)^a^
20	1	A (1)	H9 (1)	Unknown	Unknown	Mt. Yuwan, Amamiohshima Island, Kagoshima pref., Japan	Ishikawa et al. (2006)^a^
21	1	D (1)	H8 (1)	30°44′ N, 130°59′ E	22	Tanega‐shima Island, Kagoshima pref., Japan	Ishikawa et al. (2006)^a^
22	1	A (1)	ND	38°18′ N, 141°33′ E	62	Kinkazan Island, Miyagi pref., Japan	Ishikawa et al. (2006)^a^
23	2	A (1)	H8 (1)	34°17′ N, 132°18′ E	22	Miyajima Island, Hiroshima pref., Japan	Ishikawa et al. 2006)^a^
24	1	A (1)	H4 (1)	43°03′ N, 141°18′ E	34	Sapporo city, Hokkaido pref., Japan	Ishikawa et al. (2006)^a^
25	1	A (1)	H4 (1)	Unknown	Unknown	Akita pref., Japan	Ishikawa et al. (2006)^a^
26	1	A (1)	H4 (1)	38°20′ N, 141°30′ E	364	Mt. Hikariyama, Miyagi pref., Japan	Ishikawa et al. (2006)^a^
27	1	A (1)	H1 (1)	35°42′ N, 139°45′ E	23	Hongo campus of the University of Tokyo, Tokyo, Japan	Ishikawa et al. (2006)^a^
28	1	B (1)	H1 (1)	35°18′ N, 139°32′ E	8	Kamakura, Kanagawa pref., Japan	Ishikawa et al. (2006)^a^
29	1	C (1)	ND	36°35′ N, 137°26′ E	474	Toyama pref., Japan	Ishikawa et al. (2006)^a^
30	1	E (1)	H5 (1)	Unknown	Unknown	Mt. Ontake, Nagano pref., Japan	Ishikawa et al. (2006)^a^
31	1	A (1)	ND	Unknown	2200	Nagano pref., Japan	Ishikawa et al. (2006)^a^
32	1	A (1)	H1 (1)	36°35′ N, 137°27′ E	977	Toyama pref., Japan	Ishikawa et al. (2006)^a^
33	1	A (1)	H7 (1)	34°57′ N, 137°09′ E	48	Okazaki, Aichi pref., Japan	Ishikawa et al. (2006)^a^
34	1	A (1)	ND	34°50′ N, 137°32′ E	140	Mt. Maruyama, Aichi pref., Japan	Ishikawa et al. (2006)^a^
35	1	A (1)	ND	34°29′ N, 136°51′ E	66	Sakatejima Island, Mie pref., Japan	Ishikawa et al. (2006)^a^
36	1	A (1)	H8 (1)	34°32′ N, 134°59′ E	2	Awajishima Island, Hyogo pref., Japan	Ishikawa et al. (2006)^a^
37	1	A (1)	ND	Unknown	800	Mt. Daisen, Tottori pref., Japan	Ishikawa et al. (2006)^a^
38	1	A (1)	H8 (1)	35°18′ N, 133°40′ E	516	Hiruzen, Okayama pref., Japan	Ishikawa et al. (2006)^a^
39	1	A (1)	ND	Unknown	1270	Mt. Karasugasen, Tottori pref., Japan	Ishikawa et al. (2006)^a^
40	1	A (1)	H8 (1)	33°37′ N, 130°25′ E	3	Kyushu University, Fukuoka pref., Japan	Ishikawa et al. (2006)^a^
41	1	D (1)	ND	31°35′ N, 130°36′ E	7	Sakurajima Island, Kagoshima pref., Japan	Ishikawa et al. (2006)^a^
42	1	A (1)	H8 (1)	31°33′ N, 130°42′ E	33	Sakurajima Island, Kagoshima pref., Japan	Ishikawa et al. (2006)^a^
43	1	A (1)	H1 (1)	Unknown	Unknown	Tawulun Fort, Keelung, Taiwan	Ishikawa et al. (2006)^a^
44	1	A (1)	H1 (1)	Unknown	Unknown	Ilan, Taiwan	Ishikawa et al. (2006)^a^
45	1	A (1)	ND	Unknown	1700	Mt. Halla, Cheju Island, Korea	Ishikawa et al. (2006)^a^
*P. asiatica* var. *densiuscula* f. *yakusimensis*							
46	2	F (1)	H8 (1)	30°20′ N, 130°30′ E	1911	Mt. Miyanoura, Yakushima Island, Kagoshima pref., Japan	Ishikawa et al. (2006)^a^
47	1	F (1)	H8 (1)	30°19′ N, 130°30′ E	1789	Mt. Kuromi, Yakushima Island, Kagoshima pref., Japan	Ishikawa et al. (2006)^a^
*P. asiatica*							
48	1	J (1)	H11 (1)	Unknown	Unknown	China, cult.	Rønsted et al. (2002)^a^ and Iwanycki Ahlstrand et al. (2019), Kew DNA bank ID = 9585, K
*P. major*							
49	1	I (1)	Not included	Unknown	Unknown	Sachsen‐Anhalt, Germany	Rønsted et al. (2002)^a^
*P. major* var. *japonica*							
50	1	J (1)	ND	39°06′ N, 139°52′ E	5	Yusa‐cho, Akumi‐gun, Yamagata pref., Japan	
*P. camtschatica*							
51	1	K (1)	H12 (1)	38°43′ N, 139°40′ E	1	Tsuruoka city, Yamagata pref., Japan	
52	1	L (1)	H13 (1)	Unknown	Unknown	(Rahn 684, C)	Iwanycki Ahlstrand et al. (2019)^b^

Abbreviations: *n*, number of samples; ND, not determined.

^a^ Nucleotide sequence of the ITS region was determined in the indicated reference.

^b^ Nucleotide sequences of the ITS and chloroplast regions were determined in the indicated reference.

Data for the phylogenetic analysis based on the *SUC1* region were obtained from 11 individuals of *P. hakusanensis* , 1 individual of *P. asiatica* var. *densiuscula*, and 1 individual of *P. asiatica* var. *densiuscula* f. *yakusimensis*. The North American putative tetraploid *P. rugelii* Decne. was added as an outgroup. Data for the phylogenetic analysis based on ITS sequences were obtained from 25 individuals of *P. hakusanensis* , 48 individuals of *P. asiatica* var. *densiuscula*, and *P. asiatica* var. *densiuscula* f. *yakusimensis* (including 38 previously reported individuals; Ishikawa et al. 2006, Table 2), and 1 individual of *P. asiatica* from China (Rønsted et al. 2002). We also included *P. camtschatica* Link., *P. major* , and *Plantago major* var. *japonica* (Franch. et Sav.) Miyabe as related taxa. Phylogenetic analysis of the MIG‐seq data was performed using five representative individuals of *P. hakusanensis* , five of *P. asiatica* var. *densiuscula*, and one of *P. asiatica* var. *densiuscula* f. *yakusimensis*. The selected samples spanned the geographical range of each taxon. MIG‐seq is a PCR‐based method that concentrates and isolates inter‐simple sequence repeat regions located mainly in the nuclear genome (Suyama and Matsuki 2015; Suyama et al. 2022). The cp phylogenetic analysis included 24 individuals of *P. hakusanensis* from the four mountains and 37 specimens of *P. asiatica* var. *densiuscula* that included plants collected from the subalpine zone on Mt. Chokai (Table 2). We also included the cp sequences of one individual of *P. asiatica* from China (Rønsted et al. 2002; Iwanycki Ahlstrand et al. 2019; Kew DNA bank ID = 9585, K) and two individuals of *P. camtschatica* as related taxa (Iwanycki Ahlstrand et al. 2019). Although the ITS analysis included *P. major* , the species was excluded from the cpDNA analysis because excessive numbers of polymorphisms were found in *P. major* relative to *P. hakusanensis* (compared with the other taxa included in this analysis).

Total genomic DNA was extracted from fresh or dried leaves using a slightly modified cetyltrimethylammonium bromide method (Murray and Thompson 1980).

### 2.2 Chromosome Observations

We checked the chromosome numbers of *P. hakusanensis* to confirm previous reports (Yamazaki 1993; Ohashi 2017). Three individuals of *P. hakusanensis* were transplanted from Mt. Chokai to a nursery at the University of Tokyo for cytological observations. Fresh root tips were pretreated in 2 mM 8‐hydroxyquinoline solution for 1 h at 20°C and then stored at 4°C for 15 h. We subsequently fixed them in Newcomer's fluid (6:3:1:1:1 isopropanol, propionic acid, petroleum ether, acetone, 1,4‐dioxane). The root tips were macerated in 1 N HCl at 60°C for 10 min, then stained with 2% lacto‐propionic orcein, and squashed for cytological observation.

### 2.3 PCR Amplification and DNA Sequencing of the *SUC1* Region

**FIGURE 1:**
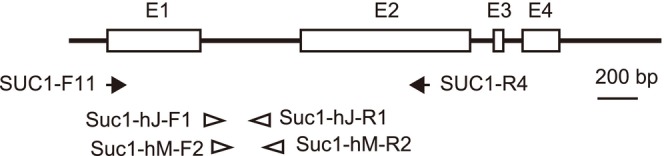
Schematic of the sucrose–proton symporter 1 (*SUC1*) gene structure and positions of the primers. Open rectangles represent exons.

Preliminary analysis indicated that the determination of the nucleotide sequence of the *SUC1* region by direct sequencing of the PCR product would be difficult, presumably because of the allotetraploid origin of *P. hakusanensis* . Thus, we applied two methods to efficiently determine two homoeologs of *SUC1* (i.e., Homoeolog L and Homoeolog I) of *P. hakusanensis* . First, the nucleotide sequences of two individuals collected from Mt. Hakusan were determined by the cloning and sequencing method described by Ishikawa et al. (2009). In this procedure, the PCR conditions were optimized to avoid over‐amplification, which potentially produces artificial recombinants among multiple alleles (homoeologs) of the polyploid (Bradley and Hillis 1997; Judo et al. 1998; Kanagawa 2003). Second, the *SUC1* sequences of nine *P. hakusanensis* individuals were obtained by homoeolog‐specific PCR amplification and direct sequencing because the cloning and sequencing method is excessively laborious. Primers for the specific PCR were designed using polymorphic sites between the homoeologs obtained by the cloning and sequencing method. Each of the two *SUC1* homoeologs (Homoeolog L and Homoeolog I) was amplified as two overlapping regions and assembled into a continuous sequence (Figure 1). For example, Homoeolog L was amplified using two primer sets: (i) SUC1‐F11 (5′‐ATGGGTGAATTGTCAGGAATTGAA‐3′) and SUC1‐hJ‐R1 (5′‐TCAAACAAATTCTGAAGTC‐3′) and (ii) SUC1‐hJ‐F1 (5′‐GATCCGTTCAATACTGATAGATCCA‐3′) and SUC1‐R4 (5′‐GAGCCACCATGTCTTAG‐3′). Homoeolog I was amplified using the following primer sets: (iii) SUC1‐F11 and SUC1‐hM‐R2 (5′‐CGATGTATACCTCTTCTATG‐3′) and (iv) SUC1‐hM‐F2: CATGGTACGGACATGGAAATGG and SUC1‐R4. The homoeolog‐specific PCR parameters were as follows: incubation at 94°C for 1 min, 20 cycles of touchdown PCR (denaturation at 94°C for 30 s, annealing at 60°C for 30 s with a temperature reduction of 0.5°C per cycle, and extension at 72°C for 1 min), 20 cycles of non‐touchdown PCR (denaturation at 94°C for 30 s, annealing at 55°C for 30 s, and extension at 72°C for 1 min), and final extension at 72°C for 7 min. PCR was performed in a 20 μL volume using TaKaRa Ex Taq polymerase (Takara Bio Inc., Shiga, Japan). The PCR products were purified with ExoSAP‐IT reagent (Thermo Fisher Scientific K. K., Tokyo, Japan) following the manufacturer's instructions. The nucleotide sequences of the PCR products were sequenced by Fasmac Co. Ltd. (Kanagawa, Japan) using the Sanger method. The GenBank accession numbers of the *SUC1* alleles are listed in Table 3. All sequencing chromatograms obtained by the Sanger method have been visually checked for quality and heterozygous sites using CLC Genomics Workbench v10.0.1 software (Filgen, Nagoya, Japan).

**TABLE 3:** List of GenBank accession numbers of the *SUC1* genotype.

SUC1	Genbank accession No. (Length)	
	Subclade I	Subclade L
Mt. Asahi_h1 (1)	LC549233 (1271 bp)	LC549245 (1250 bp)
Mt. Asahi_h2 (1)	LC549234 (1271 bp)	LC549246 (1250 bp)
Mt. Asahi_h3 (1)	LC549235 (1271 bp)	LC549247 (1250 bp)
Mt. Chokai_h4 (2)	LC549236 (1271 bp)	LC549248 (1250 bp)
Mt. Chokai_h8 (4)	LC549237 (1271 bp)	LC549249 (1250 bp)
Mt. Chokai_h19 (4)	LC549238 (1271 bp)	LC549250 (1250 bp)
Mt. Chokai_h20 (4)	LC549239 (1271 bp)	LC549251 (1250 bp)
Mt. Gassan_ (5)	LC549240 (1271 bp)	LC549252 (1250 bp)
Mt. Hakusan_h24	LC549241 (1251 bp)	ND
Mt. Hakusan_h25	LC549242 (1252 bp)	LC549253 (1237 bp)
Mt. Hakusan_16–6	LC549243 (1251 bp)	LC549255 (1250 bp)
Mt. Hakusan_5–10	LC549244 (1251 bp)	LC549254 (1250 bp)

Abbreviation: ND, not determined.

### 2.4 PCR Amplification and DNA Sequencing of the ITS and Cp Regions

The ITS region was amplified using AB101 and AB102 primers (Douzery et al. 1999). The PCR conditions were as follows: incubation at 94°C for 1 min, 25 cycles of denaturation at 94°C for 30 s, annealing at 60°C for 30 s, extension at 72°C for 1 min, followed by final extension for 7 min. Three cp regions (*trnL‐trnF*, *ndhF‐rpl32*, and *rpl32‐trnL*) were chosen based on a previous study showing that they are useful for phylogenetic studies of the subgenus *Plantago* (Dunbar‐Co et al. 2008; Iwanycki Ahlstrand et al. 2019). The cp regions were amplified using universal primers (Taberlet et al. 1991; Shaw et al. 2007). The PCR and sequencing procedures were identical to those used for the *SUC1* homoeolog‐specific PCR.

### 2.5 Phylogenetic Analysis of the *SUC1* Region

We assembled forward and reverse reads using CLC Genomics Workbench v10.0.1 software (Filgen, Nagoya, Japan). All sequence alignments were performed using MAFFT v7 software (Katoh et al. 2019) and manually edited using CLC Genomics Workbench v10.0.1 software. The phylogenetic relationships of the *SUC1* region were inferred using the maximum parsimony (MP), neighbor‐joining (NJ; Saitou and Nei 1987), and maximum likelihood (ML; Felsenstein 1981) procedures using PAUP* 4.0a software (Swofford 2003). In the MP analysis, parsimony informative indels were coded as binary (present or absent) characters, “gapmode” was set as missing, and all characters were weighted equally. The analysis was performed via a heuristic search using the tree bisection–reconnection branch‐swapping option. One hundred rounds of random additions were performed to identify multiple islands of equally most parsimonious trees (Maddison 1991). The search setting used to find MP trees was applied to 1000 bootstrap replications (Felsenstein 1985). The distance option in our NJ analysis was set to ML; the substitution rate classes and gamma shape parameter were estimated following the PAUP* procedures manual (model correspondence = GTR + G) (Swofford and Sullivan 2009; Swofford and Bell 2017). This search setting was applied to 1000 bootstrap replications. In our ML tree search, the nucleotide evolution model was selected using PAUP* 4.0a software (model correspondence = GTR + I + G) (Swofford and Sullivan 2009), and the analysis was performed via a heuristic search using the tree bisection–reconnection branch‐swapping option (addseq = random, nreps = 10). This search setting was applied to 100 bootstrap replications.

### 2.6 Median‐Joining (MJ) Network Analysis of the ITS and Cp Regions

The MJ network was constructed using PopART v1.7 software (Bandelt et al. 1999; Leigh and Bryant 2015) to determine the relationships of ITS genotypes and cp haplotypes with ε = 0. All sites with only ambiguous base states (e.g., Y, R, and K) were excluded, and indels were coded as binary (present or absent) characters. A mononucleotide repeat region detected in *rpl32‐trnL* (from 1844 bp to 1865 bp in a concatenated alignment) was removed before data analysis because of its significant homoplasy. There was a sequencing gap between 1947 bp and 1948 bp in the *rpl32‐trnL* region of the concatenated alignment because sequencing of the middle part of the *rpl32‐trnL* region was not feasible for either *P. hakusanensis* or *P. asiatica* var. *densiuscula* due to the poor quality of the raw data. The GenBank accession numbers of the ITS genotypes and cp haplotypes are listed in Tables 4 and 5, respectively.

**TABLE 4:** List of GenBank accession numbers of the ITS genotype.

Genotype of ITS	Genbank accession No.
A	AB223151.1
B	AB223153.1
C	AB223154.1
D	AB223156.1
E	AB223160.1
F	AB223162.1
G	LC549256
H	LC549257
I	AY101861
J	AY101862
K	LC549258
L	AJ548971.1

**TABLE 5:** List of GenBank accession numbers of chloroplast haplotypes.

Chloroplast haplotype (*n*)	Genbank accession no.			
	ndh‐rpl32	trnL‐trnF	rpl32‐trnL	rpl32‐trnL
			5′	3′
1	LC549259	LC549271	LC549283	LC549295
2	LC549260	LC549272	LC549284	LC549296
3	LC549261	LC549273	LC549285	LC549297
4	LC549262	LC549274	LC549286	LC549298
5	LC549263	LC549275	LC549287	LC549299
6	LC549264	LC549276	LC549288	LC549300
7	LC549265	LC549277	LC549289	LC549301
8	LC549266	LC549278	LC549290	LC549302
9	LC549267	LC549279	LC549291	LC549303
10	LC549268	LC549280	LC549292	LC549304
11	LC549269	LC549281	LC549293	LC549305
12	LC549270	LC549282	LC549294	LC549306
13	MK487875	MK487976	MK487925	

### 2.7 Preparation of the MIG‐seq Library, High‐Throughput Sequencing, and Phylogenetic Inference

In brief, we used a two‐step amplification procedure based on the protocol of Suyama and Matsuki (2015), but we changed the annealing temperature from 48°C to 38°C in the first PCR of this protocol. The amplicons were purified and sequenced on an Illumina MiSeq sequencer (Illumina, San Diego, CA, USA). Primer regions, anchors, and low‐quality reads were removed using the FASTX Toolkit package (http://hannonlab.cshl.edu/fastx_toolkit/). To remove reads derived from extremely short library entries, we searched for the sequences of the primer‐targeted regions within the sequences of reads 1 and 2, and reads containing the searched sequences were removed using TagDust software (Lassmann et al. 2009).

To obtain genotypes at SNPs, we used the Universal Network Enabled Analysis Kit (UNEAK) pipeline (Lu et al. 2013) to assemble 80‐bp clean reads. UNEAK is a non‐reference, network‐based pipeline that has been successfully used for genotype calling in polyploid species (e.g., Clark et al. 2015; Li et al. 2014). A default setting was used in the read assembly by UNEAK. SNPs were exported in HapMap format and then filtered using TASSEL 5.0 software (Bradbury et al. 2007) with the following parameters: siteMinCount 10, MinAlleleFreq 0.05, and maxHetero 0.5. The loci genotyped in all samples were retained in the final dataset.

We used RAxML v.8.2.10 software (Stamatakis 2014) to infer a ML phylogenetic tree. In this analysis, we specified the GTRGAMMA model as the nucleotide evolution model and performed 1000 bootstrap iterations to assess the node support values. The GenBank accession numbers of the MIG‐seq raw data are listed in Table 6.

**TABLE 6:** GeneBank accession numbers of MIG‐seq samples.

BioProject	BioProject submission	DRA submission	BioSample	BioSample submission	Sample name	Experiment	Experiment alias	Experiment title	Library source	Library strategy	Library layout	Run	Run alias	Run title	Run files
PRJDB9828	PSUB008116	DRA010171	SAMD00224523	SSUB014994	*Plantago hakusanensis*_NI‐1_Mt.Gassan	DRX217532	pancheri54‐0022_Experiment_0001	Illumina MiSeq sequencing of SAMD00224523	GENOMIC	AMPLICON	SINGLE	DRR227268	pancheri54‐0022_Run_0001	Illumina MiSeq sequencing of SAMD00224523	NI‐1.fastq.gz
PRJDB9828	PSUB008116	DRA010171	SAMD00224524	SSUB014994	*Plantago hakusanensis*_NI‐2_Mt.Hakusan_h24	DRX217533	Pancheri54‐0022_Experiment_0002	Illumina MiSeq sequencing of SAMD00224524	GENOMIC	AMPLICON	SINGLE	DRR227269	Pancheri54‐0022_Run_0002	Illumina MiSeq sequencing of SAMD00224524	NI‐2.fastq.gz
PRJDB9828	PSUB008116	DRA010171	SAMD00224525	SSUB014994	*Plantago hakusanensis*_NI‐3_Mt.Hakusan_h25	DRX217534	Pancheri54‐0022_Experiment_0003	Illumina MiSeq sequencing of SAMD00224525	GENOMIC	AMPLICON	SINGLE	DRR227270	Pancheri54‐0022_Run_0003	Illumina MiSeq sequencing of SAMD00224525	NI‐3.fastq.gz
PRJDB9828	PSUB008116	DRA010171	SAMD00224526	SSUB014994	*Plantago hakusanensis*_NI‐4_Mt.Chokai_h4	DRX217535	Pancheri54‐0022_Experiment_0004	Illumina MiSeq sequencing of SAMD00224526	GENOMIC	AMPLICON	SINGLE	DRR227271	Pancheri54‐0022_Run_0004	Illumina MiSeq sequencing of SAMD00224526	NI‐4.fastq.gz
PRJDB9828	PSUB008116	DRA010171	SAMD00224527	SSUB014994	*Plantago hakusanensis*_NI‐5_Mt.Chokai_h10	DRX217536	Pancheri54‐0022_Experiment_0005	Illumina MiSeq sequencing of SAMD00224527	GENOMIC	AMPLICON	SINGLE	DRR227272	Pancheri54‐0022_Run_0005	Illumina MiSeq sequencing of SAMD00224527	NI‐5.fastq.gz
PRJDB9828	PSUB008116	DRA010171	SAMD00224528	SSUB014994	*Plantago asiatica* var. densiuscula_NI‐6_Mt.Chokai_29	DRX217537	Pancheri54‐0022_Experiment_0006	Illumina MiSeq sequencing of SAMD00224528	GENOMIC	AMPLICON	SINGLE	DRR227273	Pancheri54‐0022_Run_0006	Illumina MiSeq sequencing of SAMD00224528	NI‐6.fastq.gz
PRJDB9828	PSUB008116	DRA010171	SAMD00224529	SSUB014994	*Plantago asiatica* var. densiuscula_NI‐7_Nara‐1	DRX217538	Pancheri54‐0022_Experiment_0007	Illumina MiSeq sequencing of SAMD00224529	GENOMIC	AMPLICON	SINGLE	DRR227274	Pancheri54‐0022_Run_0007	Illumina MiSeq sequencing of SAMD00224529	NI‐7.fastq.gz
PRJDB9828	PSUB008116	DRA010171	SAMD00224530	SSUB014994	*Plantago asiatica* var. densiuscula_NI‐8_Miyajima‐1	DRX217539	Pancheri54‐0022_Experiment_0008	Illumina MiSeq sequencing of SAMD00224530	GENOMIC	AMPLICON	SINGLE	DRR227275	Pancheri54‐0022_Run_0008	Illumina MiSeq sequencing of SAMD00224530	NI‐8.fastq.gz
PRJDB9828	PSUB008116	DRA010171	SAMD00224531	SSUB014994	*Plantago asiatica* f. yakusimensis_NI‐9_Mt.Miyanoura‐1	DRX217540	Pancheri54‐0022_Experiment_0009	Illumina MiSeq sequencing of SAMD00224531	GENOMIC	AMPLICON	SINGLE	DRR227276	Pancheri54‐0022_Run_0009	Illumina MiSeq sequencing of SAMD00224531	NI‐9.fastq.gz
PRJDB9828	PSUB008116	DRA010171	SAMD00224532	SSUB014994	*Plantago asiatica* var. densiuscula_NI‐10_Nara‐2	DRX217541	Pancheri54‐0022_Experiment_0010	Illumina MiSeq sequencing of SAMD00224532	GENOMIC	AMPLICON	SINGLE	DRR227277	Pancheri54‐0022_Run_0010	Illumina MiSeq sequencing of SAMD00224532	NI‐10.fastq.gz

## 3 Results

### 3.1 Chromosome Numbers

**FIGURE 2:**
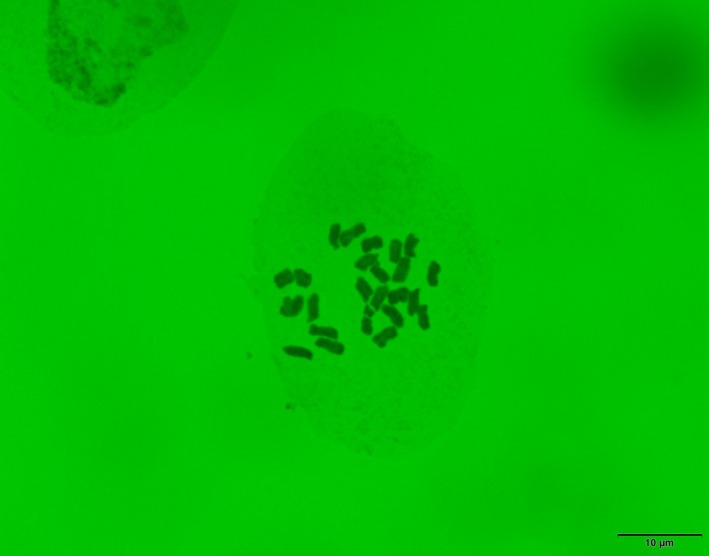
Photograph of the somatic chromosomes in a *Plantago hakusanensis* collected on Mt. Chokai.

We counted chromosome numbers of 2*n* = 24 in three individuals of *P. hakusanensis* collected on Mt. Chokai (Figure 2). This number was reported previously by Yamazaki (1993) and Ohashi (2017), but we were unable to locate original data from either study showing the collection site or photographs of chromosomes. We confirmed that the chromosome number of *P. hakusanensis* was 2*n* = 24, and that the species was a tetraploid with a basic chromosome number *x* = 6 (Rahn 1996).

### 3.2 Phylogenetic Analysis Based on *SUC1* Showed That *P. hakusanensis* is a Close Relative of *P. asiatica* var. *densiuscula* Within Sect. *Plantago*

**FIGURE 3:**
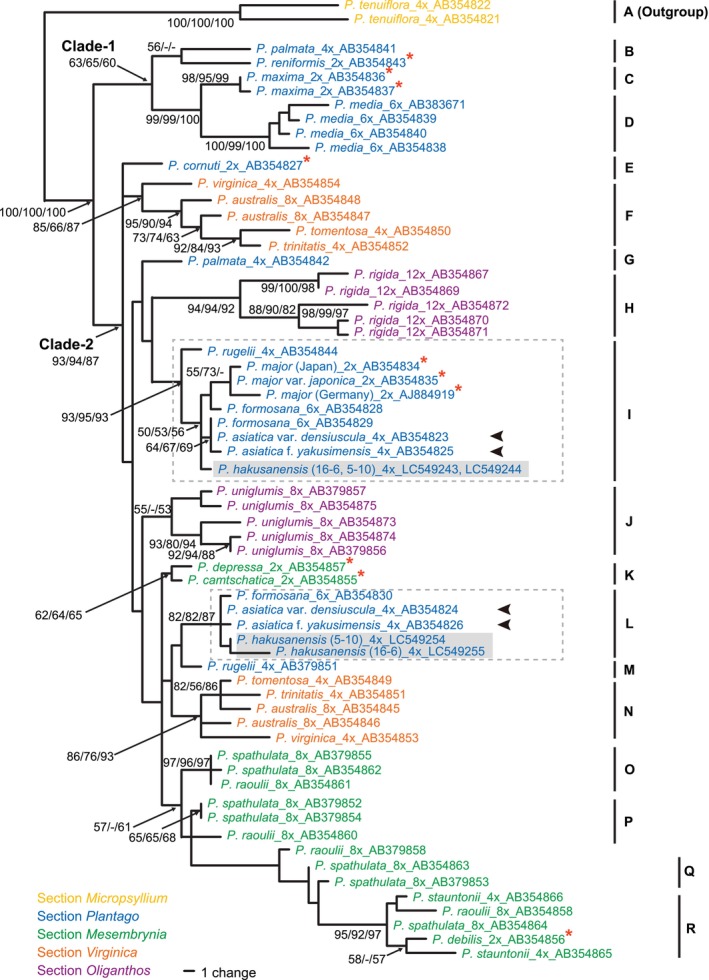
Phylogenetic position of *Plantago hakusanensis* among representative species of the subgenus *Plantago*. One of 59 most parsimonious trees based on the sucrose–proton symporter 1 (*SUC1*) sequences is presented. Bootstrap values for maximum parsimony, neighbor‐joining, and maximum likelihood analyses are located adjacent to the branches (values < 50 are shown as hyphens). The alleles of *P. hakusanensis* are enclosed in gray boxes. The alleles of *P. asiatica* var. *densiuscula* and *P. asiatica* var. *densiuscula* f. *yakusimensis* are indicated by black arrow heads. Each allele is associated with a taxon name, which is followed by an underscore, the putative ploidy level, an underscore, and the GenBank accession number. Diploid species are identified by red asterisks. Color codes for Rahn's (1996) classification of *Plantago* sections are provided in the bottom left corner.

The PCR‐amplified *SUC1* region of *P. hakusanensis* was *ca*. 1.25 kb long. Two distinct *SUC1* alleles were obtained from each of the two individuals from Mt. Hakusan. After removing the entire intron 1 from the ambiguous alignment, the aligned matrix of all 63 unique alleles from *P. hakusanensis* and 24 taxa representing all five sections of subgenus *Plantago* (2 diploids and 17 polyploids, including *P. tenuiflora* as an outgroup) was 835 bp long, with 180 variable and 111 parsimony‐informative sites. In the MP analysis, we obtained 59 most parsimonious trees with 311 steps. The overall consistency index (Kluge and Farris 1969) was 0.723, the overall retention index (Farris 1989) was 0.855, and the overall rescaled index (Farris 1989) was 0.618. The topologies produced from the MP, NJ, and ML procedures were mostly similar. The MP tree is shown in Figure 3. The NJ and ML trees are shown in Figures S1 and S2, respectively.

We compared our results with those of Ishikawa et al. (2009) and found that the addition of three *P. hakusanensis* alleles and a change in outgroup identity had little effect on the major topologies of the trees. Thus, we present below a brief summary of our results with a particular focus on *P. hakusanensis* . The alleles of the ingroups from four sections of the subg. *Plantago* fell into two sister clades: clade 1 (MP/NJ/ML support values = 63/65/60) and clade 2 (support values = 93/94/87). Three subclades (B–D) were recognized in clade 1. Three alleles (E, G, and M) and 11 subclades (F, H–L, and N–R) were found in clade 2 (Figure 3). Two alleles obtained from a *P. hakusanensis* individual were represented in the two distantly separated subclades I (support values = 93/95/93) and L (support values = 82/82/87) (Figure 3). The alleles found in the distinct subclades were considered homoeologs originating from allotetraploidization that occurred through interspecific hybridization between two diploids followed by full duplication of the hybrid genome (Ramsey and Schemske 1998; Glover et al. 2016). A set of two homoeologs in subclades I and L was also found in *P. asiatica* var. *densiuscula* and *P. asiatica* var. *densiuscula* f. *yakusimensis*; hence, *P. hakusanensis* is an allotetraploid originating from ancestral lineages shared with *P. asiatica* var. *densiuscula* and *P. asiatica* var. *densiuscula* f. *yakusimensis*.

### 3.3 Monophyly of *P. hakusanensis* Inferred by Analyses Based on *SUC1* , the ITS Region, and MIG‐seq

**FIGURE 4:**
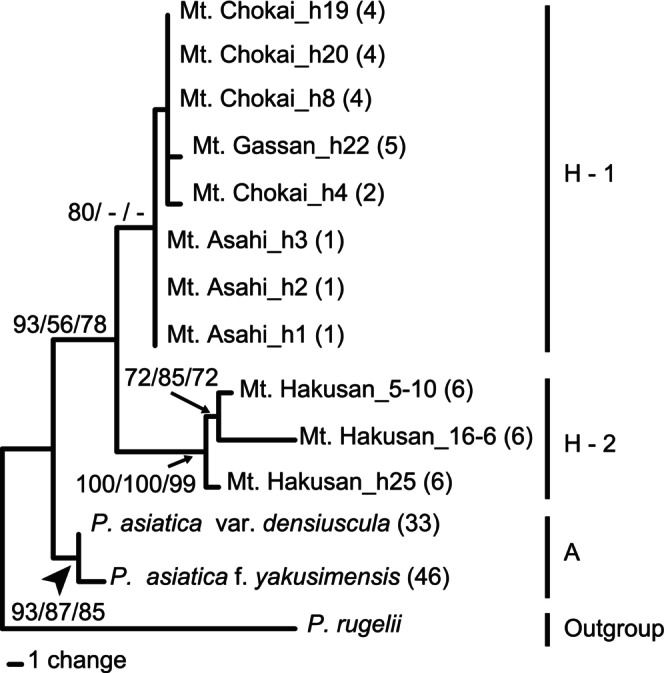
Phylogenetic tree of *Plantago hakusanensis* and *P. asiatica* var. *densiuscula* based on concatenated homoeologs of *sucrose–proton symporter 1* (*SUC1*) sequences. Bootstrap values for the maximum parsimony, neighbor‐joining, and maximum likelihood analyses are presented adjacent to the branches (values < 50 are shown as hyphens). Numbers in parentheses after each sample name refer to the “Population no.” in Table 2.

Two homoeologs of *SUC1* were successfully amplified by homoeolog‐specific PCR amplification in all 10 individuals of *P. hakusanensis* that we investigated. The homoeologs from 1250 to 1271 bp in length were successfully sequenced with one exception: one homoeolog in subclade L in an individual collected from Mt. Hakusan could not be sequenced, likely due to the presence of indels between two biparentally inherited alleles. The topologies of the trees inferred from each homoeolog of the I and L lineages were consistent (data not shown). We therefore concatenated the two homoeologs in the phylogenetic analysis. The aligned matrix of all 14 sequences comprised 2589 characters with 40 variable and 20 parsimony‐informative sites. In our MP analysis, we obtained nine most parsimonious trees after 63 steps. The overall consistency index was 0.952, the overall retention index was 0.935, and the overall rescaled index was 0.89. The topologies produced by MP, NJ, and ML were nearly identical, although there were a few slight differences in resolution at the branch tips. Thus, we present only the MP tree in Figure 4. *P. hakusanensis* formed a monophyletic clade with high to moderate bootstrap support values (MP/NJ/BI support values = 93/56/78). This clade was sister to a clade comprising *P. asiatica* var. *densiuscula* and *P. asiatica* f. *yakusimensis* (clade A). The *P. hakusanensis* clade was divided into two sister clades: subclade H‐1 (Mt. Chokai, Mt. Gassan, and Mt. Asahi) and subclade H‐2 (Mt. Hakusan).

**FIGURE 5:**
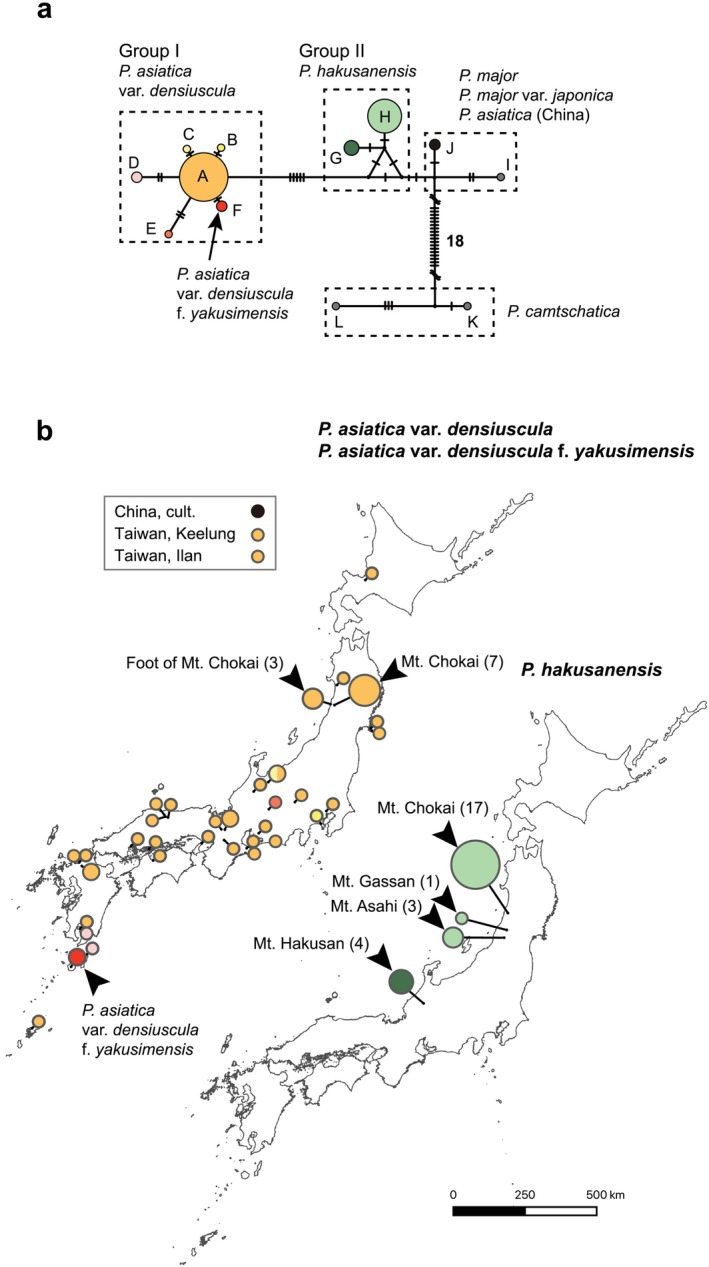
(a) Median‐joining (MJ) network of the rDNA internal transcribed spacer (ITS) genotypes of *Plantago hakusanensis* and related taxa. Black bars indicate substitutions or indels. The sizes of the circles within the MJ network are proportional to the sample size (*n*): The smallest circle represents a sample size of 1, and the largest circle for genotype A represents a sample size of 41. Unlabeled small black dots at the nodes represent inferred genotypes. (b) Geographic distributions of ITS genotypes in *P. asiatica* var. *densiuscula* and *P. hakusanensis* . The sample sizes at the representative localities are indicated in parentheses. The smallest circle represents a sample size of 1, and the largest circle for Mt. Chokai represents a sample size of 17. The sizes of the circles within the maps are proportional to the sample sizes (*n*). The colors of the circles conform to the colors in Panel (a).

Among 78 individuals examined, substitutions and indels in the ITS regions were recognized at 44 and 4 sites in 742 characters, respectively. There were 25 parsimony‐informative sites. The variable positions indicated a total of 12 genotypes (Table 7). The MJ network showed that those genotypes were divided into three taxon‐specific groups (I, II, and *P. camtschatica* ); the remaining members of the group belonged to *P. asiatica* from China, *P. major* , and *P. major* var. *japonica* (Figure 5 and Table 2). Group I contained six genotypes of *P. asiatica* var. *densiuscula*, and group II contained two genotypes of *P. hakusanensis* ; these two groups were distinctly separated by six mutation steps. Within the MJ network, group I had a star‐like configuration in which one major genotype was surrounded by several low‐frequency genotypes distinguished by one or two mutation steps.

**TABLE 7:**
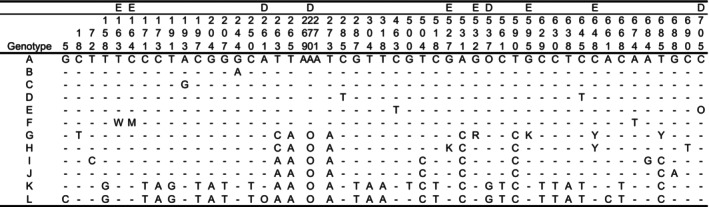
Differences found in sequences of the rDNA internal transcribed spacer (ITS) locus.

*Note:* Sequences are numbered from the 5′ end to the 3′ end. —: Same as genotype A, O: absence, D: site with indel, E: polymorphic site consisted of only an ambiguous base (e.g., W and M), and the site was excluded from the analysis.

Since the distributions of both taxa overlap along a mountain trail on Mt. Chokai at elevations between 1320 and 1540 m, we hypothesized that recent hybridization between *P. hakusanensis* and *P. asiatica* var. *densiuscula* might be detectable in ITS sequences due to the biparental inheritance of nuclear alleles. However, sequencing chromatograms indicated no sign of hybridization in the 17 individuals of *P. hakusanensis* and 7 individuals of *P. asiatica* var. *densiuscula* sampled from Mt. Chokai.

**FIGURE 6:**
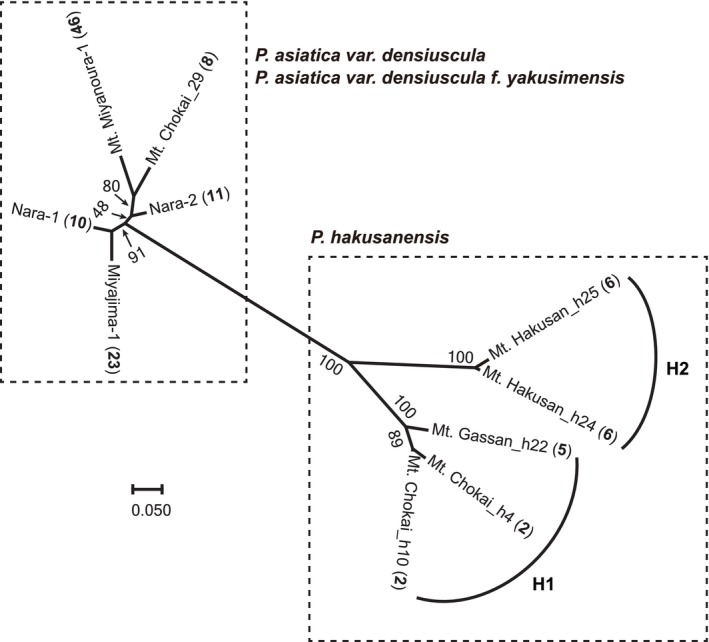
A maximum likelihood tree of *Plantago hakusanensi*s and *P. asiatica* var. *densiuscula* based on the single‐nucleotide polymorphisms obtained by MIG‐seq. Bootstrap values are presented adjacent to the branches. The scale bar represents the mean number of nucleotide substitutions per site. Boldface numbers in parentheses adjacent to the sample names refer to the “Population no.” in Table 2.

MIG‐seq analysis obtained an average of 2,561,487 reads from 10 individuals. The genotype matrix consisted of 420 SNPs, and the genotyping rate at these markers was 1.0. Phylogenetic analyses revealed the monophyly of *P. hakusanensis* (Figure 6, bootstrap value = 100). The *P. hakusanensis* clade was divided into two sister clades (H1 and H2, bootstrap value = 100). Clade H1 comprised individuals from Mt. Chokai and Mt. Gassan. Clade H2 comprised individuals from Mt. Hakusan, similar to the *SUC1* tree (Figures 4 and 6). Branch lengths from the tips to the nodes at the last common ancestor for each taxon were shorter in *P. asiatica* var. *densiuscula*, even though it has a wide distribution across the Japanese Archipelago.

### 3.4 Cp Haplotypes Shared Among *P. hakusanensis* , *P. asiatica* var. *densiuscula*, and *P. asiatica* From China

**FIGURE 7:**
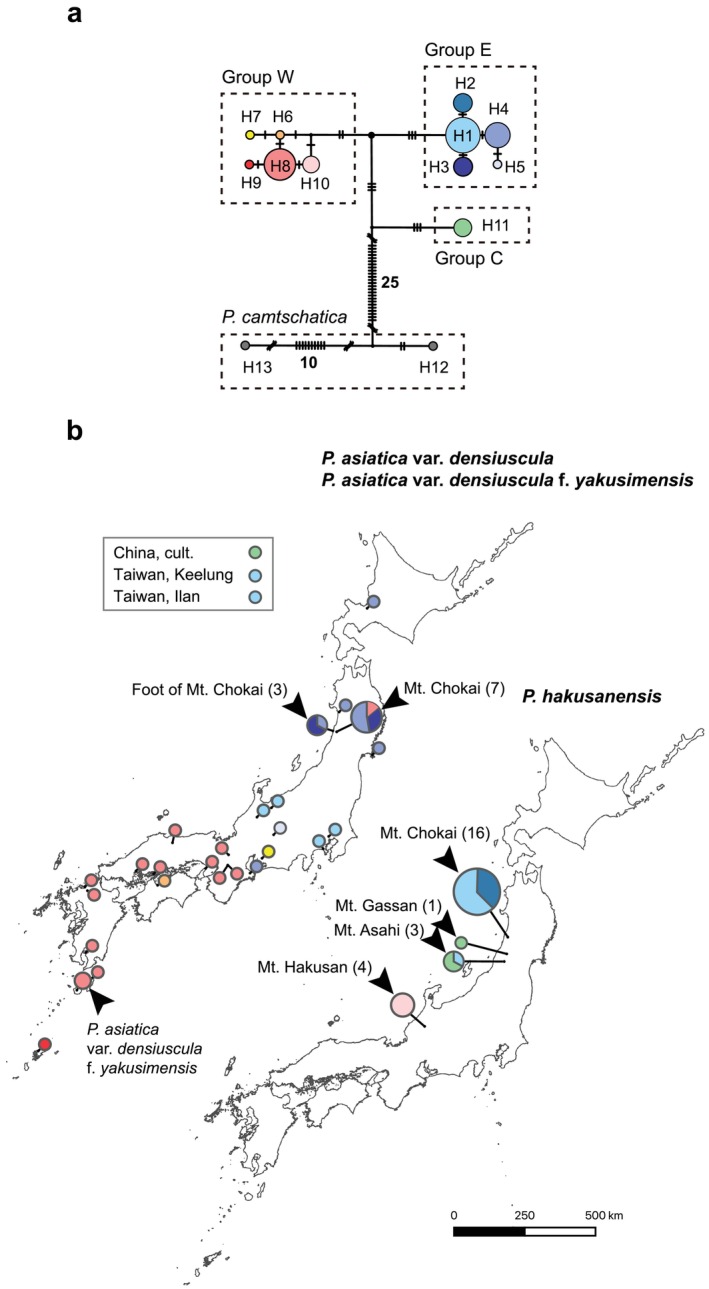
(a) Median‐joining (MJ) network of chloroplast (cp) haplotypes in *Plantago hakusanensis*, *P. asiatica* var. *densiuscula*, and *P. camtschatica* . Each black bar indicates a substitution or an indel, depending on the site. The sizes of the circles within the network are proportional to the sample size (*n*): The smallest circle represents a sample size of *n* = 1, and the largest circle for haplotype H1 represents a sample size of *n* = 17. Unlabeled small black dots at the nodes represent inferred genotypes. (b) Distribution of cp haplotypes in *P. asiatica* var. *densiuscula* and *P. hakusanensis* . Sample sizes at the representative localities are shown in parentheses. The smallest circle represents a sample size of 1; the largest circle (Mt. Chokai) represents a sample size of 16. The sizes of the circles within the maps are proportional to the sample size (*n*). The colors of the circles conform to those in Panel (a).

Among 64 individuals examined, substitutions and indels were recognized at 46 and 14 sites in 2175 characters, respectively. There were 36 parsimony‐informative sites. The variable positions indicated a total of 13 haplotypes (Table 8). Eleven haplotypes were taxon‐specific, but two haplotypes were shared by different taxa. The first shared haplotype, H1, was shared by *P. hakusanensis* and *P. asiatica* var. *densiuscula*. The second haplotype, H11, was shared by *P. hakusanensis* and *P. asiatica* in China (Table 2). MJ network analysis showed that these haplotypes were divided into four groups (E, W, C, and *P. camtschatica* , Figure 7a). *P. asiatica* var. *densiuscula* contained groups E (haplotypes H1, H3–H5) and W (haplotypes H6–H9). The distributions of the two groups in *P. asiatica* var. *densiuscula* did not overlap, except in the case of one individual on Mt. Chokai (Figure 7). Individuals with group E haplotypes were found in Taiwan and in the eastern part of the Japanese Archipelago. Group W was distributed mainly within the western part of the Japanese Archipelago (Table 2). Haplotypes of *P. hakusanensis* consisted of groups E (H1 and H2), W (H10), and C (H11). Haplotypes H2 and H10 were specific to *P. hakusanensis* , although they were separated from haplotypes H1 and H8 of *P. asiatica* var. *densiuscula*, respectively, by only one substitution (H1 was shared by both *P. hakusanensis* and *P. asiatica* var. *densiuscula*, as indicated above).

**TABLE 8:**
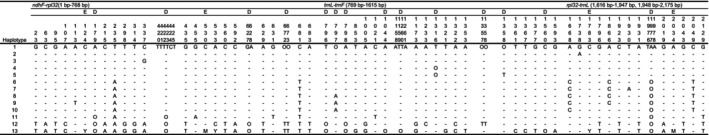
Differences found in three chloroplast loci (*ndhF‐rpl32*, *trnL‐trnF*, and *rpl32*‐*trnL*).

*Note:* Sequences are numbered from the 5′ end to the 3′ end. —: Same as haplotype 1, O: absence, D: site with indel, E: polymorphic site consisted of only an ambiguous base (e.g., Y and M), and the site was excluded from the analysis.

## 4 Discussion

### 4.1 *P. hakusanensis* is an Allotetraploid Related to *P. asiatica* var. *densiuscula*

Our phylogenetic analyses based on the nuclear‐encoded single‐copy *SUC1* region showed that *P. hakusanensis* and *P. asiatica* var. *densiuscula* (and *P. asiatica* var. *densiuscula* f. *yakusimensis*) are both allotetraploids with two distinctly related homoeologs. Each homoeolog was found to belong to the same subclade with high support values (subclade I and L in Figure 3). Thus, *P. hakusanensis* and *P. asiatica* var. *densiuscula* are closely related to one another, and *P. hakusanensis* is presumed to have originated either from the same ancestral allotetraploid as *P. asiatica* var. *densiuscula*, with subsequent differentiation into distinct taxa in different biomes, or from independent allopolyploidization via hybridization between the same or closely related parental species of *P. asiatica* var. *densiuscula*.

A diploid *P. major* (or *P. major* var. *japonica*) in subclade I and a diploid species related to *P. depressa* and *P. camtschatica* in sect. *Mesembrynia* (subclade K, Figure 3) have been proposed to be the parental species in the allopolyploidization of *P. asiatica* var. *densiuscula*, based on their phylogenetic positions and current distributions (Ishikawa et al. 2009). The assumptions made for *P. asiatica* var. *densiuscula* would also be applicable to the parental species of *P. hakusanensis* . In contrast to our *SUC1* analysis results, only one lineage of ITS sequences was obtained from both *P. asiatica* var. *densiuscula* and *P. hakusanensis* ; these sequences were closely related to *P. major* and *P. major* var. *japonica* (Figure 5). This result may be explained by the failure of PCR to amplify ITS sequences related to *Plantago camtschatica*, or biparentally inherited homoeologous ITS regions might have been homogenized by both inter‐locus and intra‐locus concerted evolution biased toward one of the two parental lineages ( *P. major* or *P. major* var. *japonica*), as reported in other allopolyploids (Wendel et al. 1995; Kovarik et al. 2005). The former possibility may be less likely because the ITS region of *P. camtschatica* was successfully amplified in the present study, and the primer set used in this analysis has been shown to be appropriate for a wide taxonomic range (Douzery et al. 1999; Sonboli et al. 2011; Kokubugata et al. 2011; Koecke et al. 2013).

### 4.2 Disparities Between Phylogenies Based on Nuclear‐ and Cp‐Encoded Genes in *P. hakusanensis* and *P. asiatica* var. *densiuscula*

Phylogenetic analyses of the two nuclear coding regions (*SUC1* and *ITS*) and MIG‐seq data revealed the monophyly of *P. hakusanensis* , at least in the samples examined in our study (Figures 3, 4, 5, 6). In contrast, cp haplotypes of *P. hakusanensis* (haplotypes H1, H2, H10, and H11) were shared or phylogenetically close to *P. asiatica* var. *densiuscula* and *P. asiatica* in China. In general, shared genetic diversity between closely related taxa is explained by incomplete lineage sorting of ancestral polymorphisms and/or introgression (Rieseberg and Soltis 1991; Comes and Abbott 2001; Dixon et al. 2007). Although it is difficult to rule out the possibility of incomplete lineage sorting completely, the shared and related haplotypes in *P. hakusanensis* and *P. asiatica* var. *densiuscula* and *P. asiatica* may be best explained by introgressions of the cp genome (cp capture) for the three reasons outlined below. First, incomplete lineage sorting tends to occur shortly after speciation, but we consider the two taxa to be distinctly differentiated from one another based on the results of nuclear marker analyses (Figures 4, 5, 6). Second, major haplotype groups (W, E, and C; Figure 5) were also distinctly differentiated from one another (W, E, and C; Figure 5). Third, ancestral polymorphisms in incomplete lineage sorting are more readily fixed in the cp genome, which has a smaller effective population size than that of the nuclear genome (Schaal et al. 1998), although we detected shared genetic diversity between taxa in the cp markers examined in the present study.

We found the cp haplotypes of *P. asiatica* var. *densiuscula* groups W and E in the western and eastern parts of the Japanese Archipelago, respectively (Figure 7). A similar geographic structure also occurred in *P. hakusanensis* . Although the direction of cp genome introgression between *P. asiatica* var. *densiuscula* and *P. hakusanensis* is unclear, we consider two possible hypotheses below. First, the geographic structure was originally established in *P. asiatica* var. *densiuscula*, and two independent cp genome introgressions from *P. asiatica* var. *densiuscula* to *P. hakusanensis* occurred on Mt. Hakusan (group W) and in the Tohoku area (group E). Similar geographic distribution patterns of nuclear genotypes and/or cp haplotypes have been reported to be common in many other plants in temperate forests (Iwasaki et al. 2012), and the geographic patterns are assumed to have been formed by the isolation of ancestral populations into different refugia during the Quaternary glacial climate and postglacial expansions (Aoki et al. 2019; Sakaguchi et al. 2021). Alternatively, the second hypothesis proposes that haplotypes in groups W and E originated from *P. asiatica* var. *densiuscula* and *P. hakusanensis* , respectively. Both empirical and simulation studies have reported that neutral gene introgression tends to occur from locally established species to invading taxa (Currat et al. 2008; Excoffier et al. 2009). Studies of the genera *Veratrum* and *Cercidiphyllum* also hypothesized ancient introgression of the cp genome from local species in subalpine cool‐temperate forest habitats to invading species in low‐ to mid‐elevation warm‐temperate locations (Kikuchi et al. 2010; Qi et al. 2012). Under this hypothesis, *P. hakusanensis* may have differentiated in central Honshu, with cp genome (group E) introgression subsequently occurring from local *P. hakusanensis* to invading *P. asiatica* var. *densiuscula*. *P. asiatica* var. *densiuscula* would have rapidly expanded its distribution range from the southwestern Japanese Archipelago toward the northeast because, within the median‐joining network (Figure 5), ITS group I had a star‐like configuration, in which one major genotype was surrounded by several low‐frequency genotypes distinguished by one or two mutation steps, which is indicative of rapid population expansion. Moreover, the MIG‐seq maximum parsimony tree shows shorter branch lengths from the tips to the last common ancestor for *P. asiatica* var. *densiuscula* (Figure 6), despite its broad distribution. This result also implies that *P. asiatica* var. *densiuscula* expanded more rapidly than *P. hakusanensis* . These findings support the second hypothesis; however, under this scenario, an additional cp genome introgression (group W) from *P. asiatica* var. *densiuscula* to *P. hakusanensis* on Mt. Hakusan would be required to account for the current haplotype distribution at least (Figure 7).

## Author Contributions

**Naoko Ishikawa:** conceptualization (equal), formal analysis (lead), funding acquisition (lead), writing – original draft (lead). **Shota Sakaguchi:** formal analysis (supporting), writing – original draft (supporting), writing – review and editing (equal). **Chikako Hasekura:** investigation (equal). **Alexey Shipunov:** writing – review and editing (equal). **Ayumi Matsuo:** investigation (equal). **Yoshihisa Suyama:** writing – review and editing (equal). **Hirokazu Tsukaya:** writing – review and editing (equal). **Hiroshi Ikeda:** conceptualization (equal), writing – review and editing (equal). **Motomi Ito:** writing – review and editing (equal).

## Conflicts of Interest

The authors declare no conflicts of interest.

## Supporting information


Figure S1.

Figure S2.

## Data Availability

The DNA sequences generated in this study have been deposited in the National Center for Biotechnology Information, and the accession numbers are listed in Tables 3, 4, 5, 6.
