# Supplementary figures and images for: Allotetraploid Origin and Putative Ancient Introgression in Plantago hakusanensis (Plantaginaceae)
Source: Ecol Evol. 2025 Mar 17;15(3):e71144. doi: 10.1002/ece3.71144 (PMC11917114; doi:10.1002/ece3.71144)

NJ tree

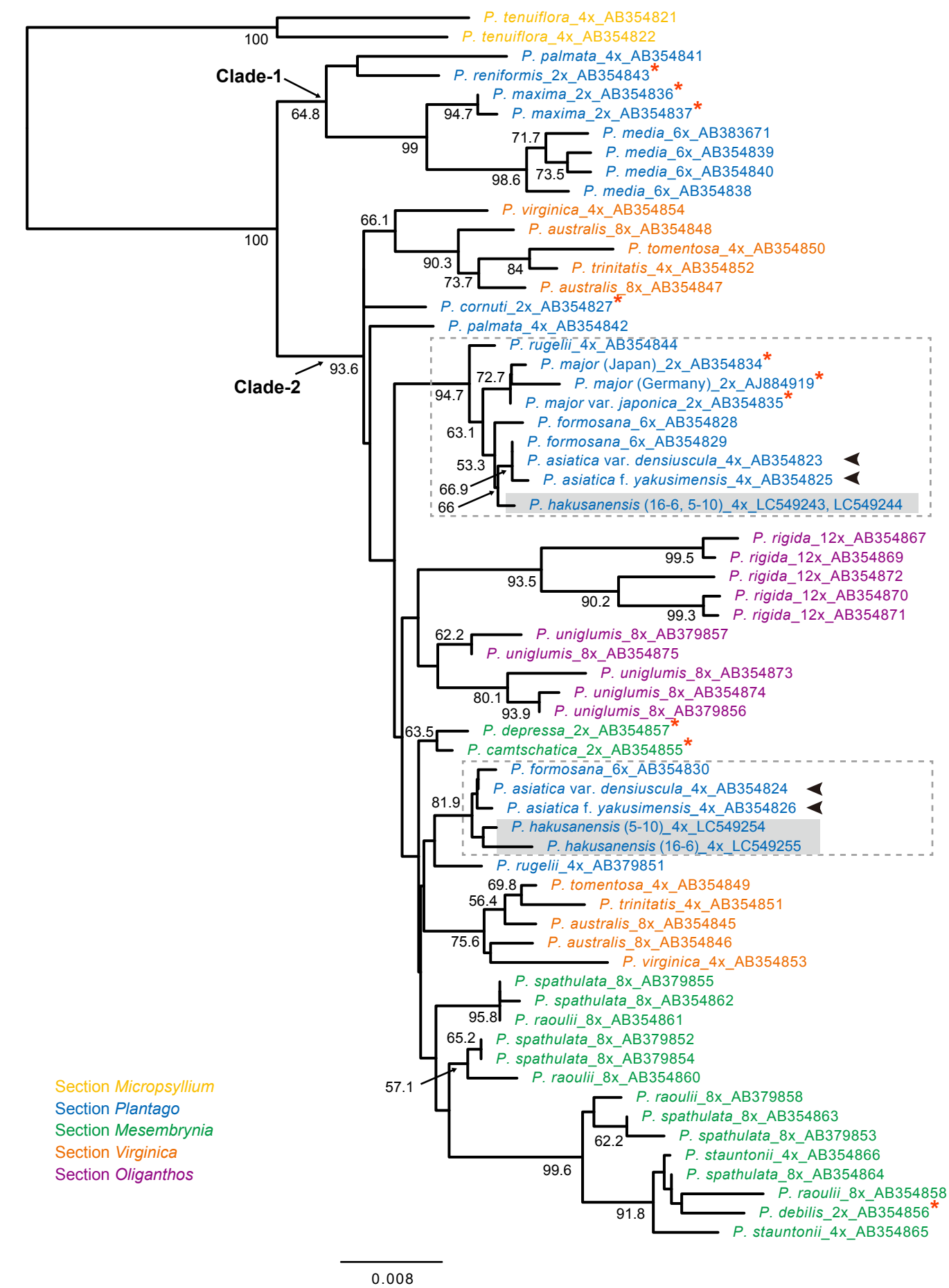

Supplement: Supplementary file 1 — Figure S1. [file ECE3-15-e71144-s001.pdf]

ML tree

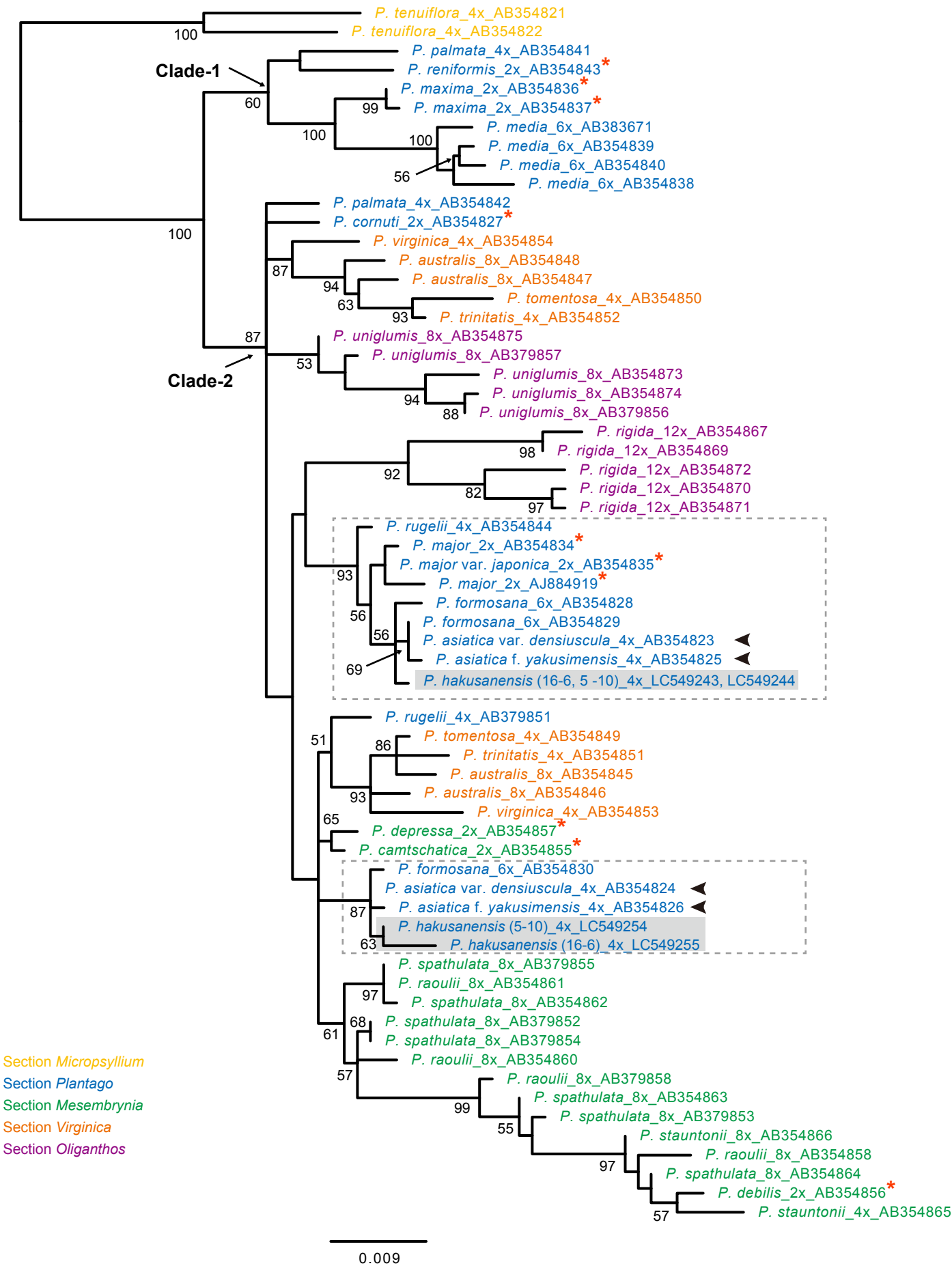

Supplement: Supplementary file 2 — Figure S2. [file ECE3-15-e71144-s002.pdf]
